# Does Protein Supplementation Support Adaptations to Arduous Concurrent Exercise Training? A Systematic Review and Meta-Analysis with Military Based Applications

**DOI:** 10.3390/nu13051416

**Published:** 2021-04-23

**Authors:** Shaun Chapman, Henry C. Chung, Alex J. Rawcliffe, Rachel Izard, Lee Smith, Justin D. Roberts

**Affiliations:** 1 HQ Army Recruiting and Initial Training Command, UK Ministry of Defence, Upavon, Wiltshire SN9 6BE, UK; alex.rawcliffe103@mod.gov.uk; 2 Cambridge Centre for Sport and Exercise Sciences, School of Psychology and Sport Science, Anglia Ruskin University, East Road, Cambridge CB1 1PT, UK; henry.chung@pgr.anglia.ac.uk (H.C.C.); lee.smith@aru.ac.uk (L.S.); justin.roberts@aru.ac.uk (J.D.R.); 3 Defence Science and Technology, Porton Down, UK Ministry of Defence, Salisbury, Wiltshire SP4 0JQ, UK; rachel.izard715@mod.gov.uk

**Keywords:** protein supplementation, training, exercise, adaptations, concurrent training

## Abstract

We evaluated the impact of protein supplementation on adaptations to arduous concurrent training in healthy adults with potential applications to individuals undergoing military training. Peer-reviewed papers published in English meeting the population, intervention, comparison and outcome criteria were included. Database searches were completed in PubMed, Web of science and SPORTDiscus. Study quality was evaluated using the COnsensus based standards for the selection of health status measurement instruments checklist. Of 11 studies included, nine focused on performance, six on body composition and four on muscle recovery. Cohen’s d effect sizes showed that protein supplementation improved performance outcomes in response to concurrent training (ES = 0.89, 95% CI = 0.08–1.70). When analysed separately, improvements in muscle strength (SMD = +4.92 kg, 95% CI = −2.70–12.54 kg) were found, but not in aerobic endurance. Gains in fat-free mass (SMD = +0.75 kg, 95% CI = 0.44–1.06 kg) and reductions in fat-mass (SMD = −0.99, 95% CI = −1.43–0.23 kg) were greater with protein supplementation. Most studies did not report protein turnover, nitrogen balance and/or total daily protein intake. Therefore, further research is warranted. However, our findings infer that protein supplementation may support lean-mass accretion and strength gains during arduous concurrent training in physical active populations, including military recruits.

## 1. Introduction

Concurrent training is defined as the combination of resistance and endurance training as part of a periodised physical training model [1]. The simultaneous development of strength, power and endurance is required by many athletic and exercising populations to meet the physical demands of their chosen sporting discipline (e.g., soccer, rugby, hockey) or exercise activity (e.g., circuits, cross-fit training) [2,3,4,5,6]. Similarly, recruits undergoing arduous military training routinely engage in concurrent training so as to meet the training and operational demands of military life [7,8,9,10,11,12,13]. Military recruit training programmes are designed to transform civilians into trained soldiers, therefore, physical training is necessarily arduous, involving a combination of aerobic training, strength and conditioning, obstacle courses, swimming, circuit training and loaded marching [14,15]. Despite the requirement of concurrent training in athletic and military recruit populations, and the positive effects protein supplementation may have on training outcomes, the majority of systematic reviews and meta-analyses have focused mainly on the effects of protein supplementation when either resistance or endurance training are studied in isolation with no specific population in particular being studied [16,17,18,19].

In untrained individuals, a bout of endurance exercise upregulates muscle protein synthesis (MPS) in mitochondrial and myofibrillar proteins, whereas, a bout of resistance training elicits an increase primarily in myofibrillar protein synthesis [20]. Moreover, a period of chronic (10 weeks) endurance or resistance training refines the MPS response following exercise in proteins specific to each mode of training. Resultantly, chronic endurance training improves the oxidative capacity of muscle, which can increase whole-body oxygen uptake, leading to a more fatigue-resistant muscle, whereas resistance training develops muscle strength [21]. Both modes of exercise have been shown to increase the phosphorylation of protein in the protein kinase B-mammalian target of rapamycin-p70 ribosomal protein S6 kinase (Akt-mTOR-p70S6K) pathway, leading to an increase of MPS [20]. Indeed, studies have suggested an interference effect when both modes of training are conducted concurrently within the same training programme [22,23,24,25,26,27,28], however, others have disputed this interaction [29,30,31]. Mechanistically, endurance exercise stimulates a rise in adenosine monophosphate-activated protein kinase (AMPK) [32], which may inhibit mTOR through the activation of the tuberous sclerosis complex (TSC) [2]. This has the potential to reduce the post-exercise MPS response, and subsequently attenuate muscle strength adaptations [27] when individuals undertake concurrent endurance training [28]. 

MPS has been shown to be maximised when protein is consumed in 20–40 g doses immediately-post resistance training [33]. Studies have also shown that concurrent resistance and aerobic training stimulates myofibrillar protein synthesis to a similar degree compared to when resistance training is performed in isolation [34]. This response is further augmented when 25 g of protein is ingested in the immediate post-exercise period [35]. Elevated levels of amino acids in the blood upregulate the localisation and activation of mTOR by deactivating the TSC [27]. The concept of “nutrient sensing” has also been suggested, whereby other proteins such as VPS34 may be key at stimulating the mTOR pathway and myofibrillar protein synthesis in response to elevated blood amino acid concentrations [27,36,37]. As such, an elevated protein intake during arduous concurrent training may be an effective strategy for attenuating the interference effect of endurance exercise [27,28], by maximising mTOR activity and the MPS response to resistance training [33,38,39,40,41,42,43], thus supporting muscle strength adaptations. Moreover, individuals undertaking arduous concurrent training with limited recovery time between exercise sessions (i.e., military recruit training) may benefit further from strategies which elevate the amount of energy and protein in the diet to support muscle adaptations [44]. In addition to muscle endurance, military recruits are required to pass strength-based tests during basic training [7,8]. Therefore, strategies which support the development of muscle strength and/or attenuate the interference effect are likely to be advantageous, particularly when considering strength is a key determinant of occupational performance [7]. 

To our knowledge, no study has systematically evaluated the literature to establish the effects of protein supplementation on training adaptations during arduous concurrent training. Therefore, the aim of this systematic review and meta-analysis was to evaluate the literature on protein supplementation and its effects on adaptations to arduous concurrent exercise training in healthy individuals with potential applications to recruits undergoing military training. 

## 2. Materials and Methods

This systematic review was completed in accordance with the preferred reporting items for systematic reviews and meta-analyses (PRISMA) statement [45].

### 2.1. Eligibility Criteria

This review sought peer-reviewed papers with human participants published in English with the following Population, Intervention, Comparison and Outcome (PICO) criteria being implemented to identify eligible studies [46]. The PICO was designed with the aim of the findings of this review being applied to military recruits undertaking military training. Military recruits are typically aged between 16–35 years [47], and are required to meet aerobic fitness and muscular strength test standards such as maximal strength and muscular endurance tests [8]. Studies that did not meet all the PICO were excluded from this review. 

Population: (a) stated as healthy active male or females; (b) aged between 16–35 years. 

Intervention: (c) include both endurance/aerobic training and resistance/weight training, circuit training, cross-fit training, military training but not high-intensity interval training (HIIT); (d) daily protein supplementation included but not with vitamins and/or antioxidants or through an increased intake of whole-food protein sources in the diet; (e) studies assessing body composition and/or performance were required to have ≥two sessions per week and be ≥four weeks in duration; (f) studies assessing muscle recovery were required to be <one week in duration; (g) training sessions performed at moderate or vigorous intensity (e.g., jogging, running, cycling, weight training) [17].

Comparison: (h) changes in outcome measures across repeated timepoints; (i) participants grouped by supplement condition.

Outcome: (j) change in primary variable(s): maximal oxygen uptake ($\dot{V}$O_2max_), time trial (TT), one-repetition maximum (1RM), fat-free mass (FFM), fat mass, musculoskeletal injury (MSKI) incidence, muscle function/soreness/damage. 

### 2.2. Search Strategy and Study Selection

**Figure 1:**
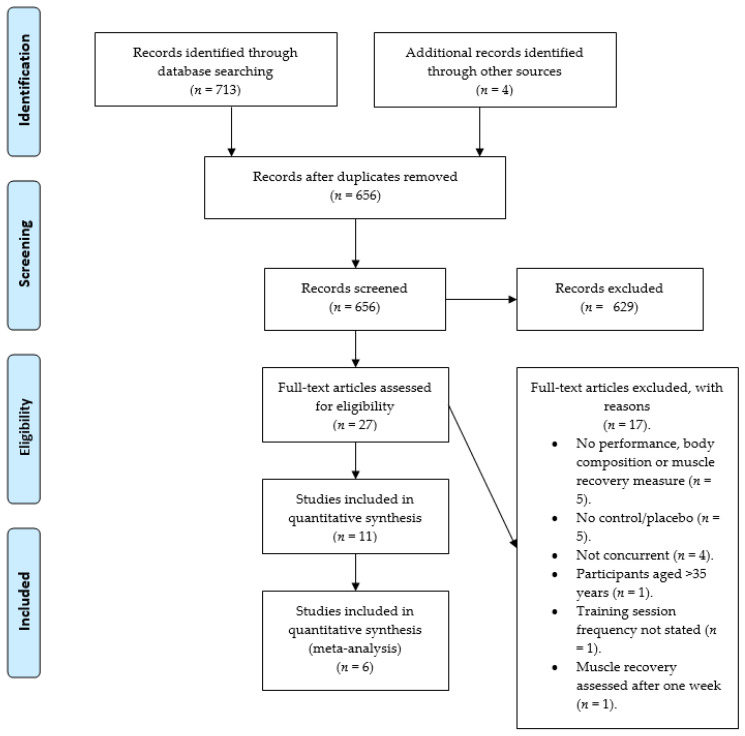
Flow chart of study retrieval process.

The final electronic database searches were completed in February 2021 in three databases (PubMed, Web of science and SPORTDiscus) using the terms “protein” or “protein supplementation”, “training”, and “concurrent training”” either alone or concurrently. The reference lists of all papers that met the inclusion criteria were interrogated to identify additional studies not found in the electronic search, until no further studies could be identified [48]. First, the study title and abstract were screened by one reviewer (SC) followed by the full text by the same reviewer. The characteristics that were extracted from each study included: author, participant sample, total protein intake (g·kg^−1^·day^−1^), training intervention, protein timing and dose. The study selection process is outlined in Figure 1. 

### 2.3. Risk of Bias Assessment

Studies were evaluated for methodological quality according to the COnsensus based standards for the selection of health status measurement instruments (COSMIN) checklist by two separate reviewers (SC and HC) (Table 1). This review used the recommended “worst score counts” method to obtain a total score for study quality. This was done by obtaining a quality score per measurement by taking the lowest rating of any item in a criteria box [49]. Each COSMIN item for all categories were scored from 4–1, where 4 was low risk and 1 was high risk. Each study needed a mean score of ≥ 3 to be included in this review [49]. 

### 2.4. Data Synthesis and Analyses

The analysis was conducted by first extracting the relevant information from all study groups at baseline and at the end of the intervention. This included the number of participants (*n*), *p* values, mean, standard deviation (SD) and 95% confidence intervals (if available). To compare the effects of protein supplementation against placebo conditions, pooling was used of the continuous data as standardized mean difference (SMD) represented as Cohen’s d effect sizes (ES), standard error (SE) and 95% confidence intervals calculated for each main outcome using the reported mean change differences (delta scores), *n* and corresponding SDs [17,19]. If the mean change difference was not reported this was calculated based on the reported pre-and-post mean and SDs in each study. If a study used multiple protein-supplemented groups, we combined the data into an overall protein-supplemented group for subsequent analyses [17]. When a study used multiple performance outcome measures, the relative $\dot{V}$O_2peak_ and lower body 1RM were prioritised for muscle strength and aerobic endurance [17]. Effect sizes were classed as small (0.2), medium (0.5), and large effects (0.8) [50]. Effect size was calculated using the following equations:

E1: Cohen’s d = (M2 − M1)/SD_pooled_

E2: SD_pooled_ = √((SD_1_^2^ + SD_2_^2^)/2)

A random-effects model was applied with heterogeneity across studies tested using I^2^ test. I^2^ values of 25%, 50%, and 75% were considered low, moderate and high, respectively [17]. Each study was weighted (%) based on its inverse within study variance and between study variance using the Meta-Essentials spreadsheet 1.4 (Microsoft Excel 2016, Washington, DC, USA). Meta-Essentials was used for the meta-analysis, creation of forest and Egger’s funnel plots (including the trim and fill method) and running statistical analysis, with alpha set at *p* ≤ 0.05. 

## 3. Results

### 3.1. Study Quality and Risk of Bias Assessment 

Table 1 outlines the quality assessment scores for each study. All 11 studies were considered eligible for this review based on their COSMIN quality assessments scores. 

**Figure 2:**
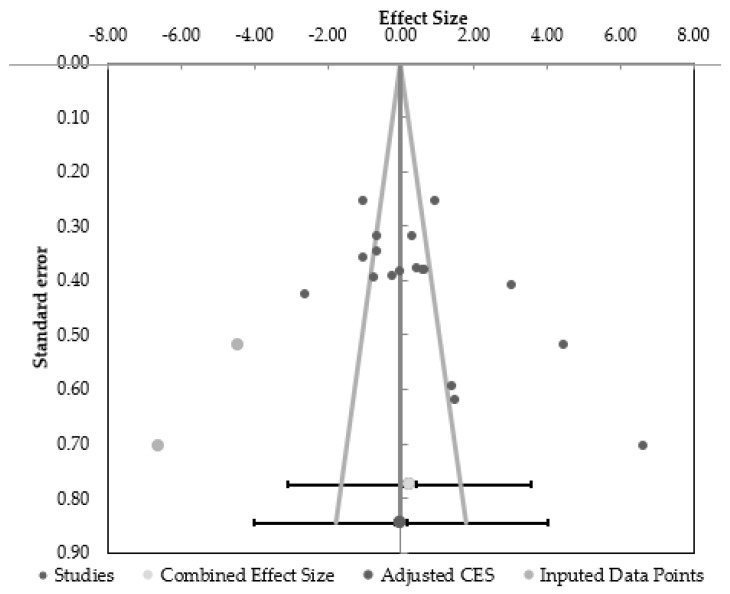
Funnel plot of the comparison of the effect of protein supplementation vs. placebo on muscle strength, aerobic endurance, fat-free mass (FFM) and fat-mass (FM) adaptations. CES = combined effect size.

Egger’s regression analysis found asymmetries in the funnel plot (Figure 2) suggesting that results might be influenced by biasing factors such as publication bias. One study was particularly responsible for this asymmetry [57] as they showed the strongest beneficial effects of the treatment group when compared with the placebo group. When this outlier was removed the funnel plot was symmetric (*p* > 0.05). The funnel plot with this outlier included can be seen in Supplementary Figure S1. 

### 3.2. Participant Characteristics and Study Interventions

Details of the studies’ characteristics are provided in Table 2 and Table 3. The sample sizes ranged from 10 to 387 with a total participant sample size of 681 (645 men, 61 women) for all studies. The reported mean age of participants ranged between 18 and 31 years. Intervention durations ranged from one day to six months with seven studies using a standardised concurrent endurance and resistance training programme [53,54,55,56,57,58], two studies using a military training programme [51,58] and two studies using an acute loaded march protocol [59,60,61]. 

### 3.3. Protein Dose and Timing

The majority of studies supplemented participants with whey protein in the form of a beverage, except one which used a protein bar [59], with the most common strategy being to provide an absolute bolus dose of protein ranging between 20–50 g. One study provided an additional dose of whey protein relative to body mass (2.4 g·kg^−1^·day^−1^) [54]. Crowe et al. [53] provided participants with a leucine supplement (45 mg·kg^−1^·day^−1^). In terms of timing, the majority of studies provided protein immediately (<1 h) post-exercise [52,55,56,57,58,59,60,61]. Others also provided protein at breakfast [54] and some provided protein both prior to sleep and immediately post-exercise [51]. Seven studies assessed and reported total daily protein intake [51,52,53,54,55,57,60], whereas four studies did not [56,58,59,61]. 

### 3.4. Synthesis of Results

This review identified 11 individual studies; one focused only on performance [55], six on performance and body composition [51,53,55,56,57,58] and four on muscle recovery adaptations only [52,59,60,61]. Four studies found a benefit on muscle strength, five studies reported a benefit on body composition changes such as increasing FFM, reducing fat-mass or both, and one study reported a benefit on muscle recovery. The characteristics of these studies are outlined in Table 2 and Table 3. 

#### 3.4.1. Performance Adaptations

**Figure 3:**
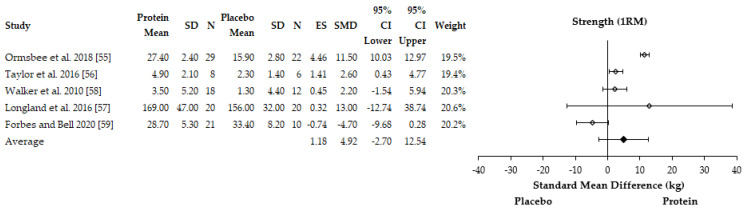
Forest plot of the studies which assessed the effects of protein supplementation on muscle strength adaptations. SD = standard deviation, *N* = sample size, ES = effect size, SMD = standard mean difference, CI = confidence interval.

**Figure 4:**
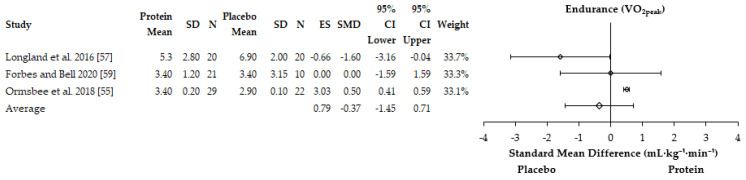
Forest plot of the studies which assessed the effects of protein supplementation on aerobic endurance adaptations. SD = standard deviation, *N* = sample size, ES = effect size, SMD = standard mean difference, CI = confidence interval.

McAdam et al. [51] observed a greater increase in muscle strength (push-up repetition performance) with protein supplementation (+6.8, 95%CI: 2.9–10.7) compared to a placebo (+2.6, −0.7–6.0 95% CI) during a two-minute maximal push-up test. There was no effect on run time performance (protein: −48.3 s, −63.0–33.6 s 95%; placebo: −74.2 s, −95.5–51.9 95% CI) during a two-minute maximal time trial. Similarly, Ormsbee et al. [55] found a greater increase in 1 RM bench press after six-months of concurrent training with protein supplementation (+27.4 ± 2.4 kg vs. +15.9 ± 2.8 kg, *p* = 0.003) but not 1 RM hip sled performance (protein: 72.3 ± 7.8 kg; placebo: 73.6 ± 9.0 kg). Both groups had a statistically significant increase in $\dot{V}$O_2peak_ at six months compared to baseline but no differences between groups were reported. Taylor et al. [56] reported a statistically significant change in 1 RM bench press performance with additional protein in female basketball players over an eight-week period (protein = +4.9 ± 2.1 kg vs. placebo = +2.3 ± 1.4 kg, *p* = 0.046). Walker et al. [58] reported a statistically significant increase in 1 RM bench press performance (protein: +3.5 ± 5.2 kg; placebo: +1.3 ± 4.4 kg, *p* < 0.05) and the number of push-ups (protein: +5.4 ± 6.8; placebo: +3.2 ± 6.8, *p* < 0.05) performed with protein supplementation over eight weeks of recruit military training. There was, however, no effect of protein on run time performance during a maximal three-mile time-trial (protein: −1.4 ± 0.4 s; placebo: −0.9 ± 3.3 s, *p* > 0.05). The remaining study also observed a greater increase in rowing time to exhaustion with leucine supplementation compared to a placebo (*p* = 0.008) [53]. The remaining three studies reported no statistical effects on exercise performance with protein supplementation compared to a placebo or control condition [54,57]. For instance, Longland et al. [57] reported no impact of protein supplementation on leg and bench press 1 RM or cycling time trial performance. Similarly, no differences in men or women were reported between groups for changes in $\dot{V}$O_2peak_, 2000 m rowing time trial, leg and bench press 1 RM performance [54]. It was possible to include five and three studies in the meta-analyses for muscle strength [54,55,56,57,58] and aerobic endurance adaptations [54,55,57], respectively. One study was not included in the muscle strength analysis [51] whilst two studies were not included in the aerobic endurance adaptations [51,53] due to no SD being reported. Additionally, another study was removed from the $\dot{V}$O_2peak_ meta-analysis due to assessing time trial performance [58]. The results of the meta-analysis are reported as SMD and showed that protein supplementation improved performance outcomes when muscle strength and aerobic endurance parameters were analysed together (SMD = 0.89, 95% CI = 0.08–1.70). When performance outcomes were analysed independently, protein supplementation was found to enhance muscle strength adaptations during concurrent training compared to placebo (ES = 1.18, SMD = +4.92 kg, 95% CI = −2.70–12.54 kg) (Figure 3). However, the meta-analysis found protein supplementation to not enhance aerobic endurance adaptations ($\dot{V}$O_2peak_) with the analysis favoring placebo (ES = 0.79, SMD = −0.37 ml·kg^−1^·min^−1^, 95% CI = −1.45–0.71) (Figure 4). The individual study effect sizes for muscle strength and aerobic endurance adaptations can be found in Supplementary Figures S2 and S3. There was substantial heterogeneity between studies for muscle strength (I^2^ = 94%) and aerobic endurance adaptations (I^2^ = 95%). 

#### 3.4.2. Body Composition Adaptations

**Figure 5:**
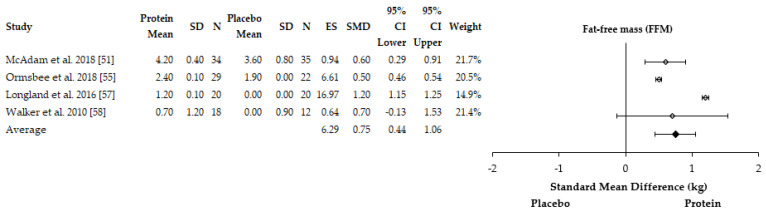
Forest plot of the studies which assessed the effects of protein supplementation on fat-free mass (FFM) changes in response to concurrent training. SD = standard deviation, *N* = sample size, ES = effect size, SMD = standard mean difference, CI = confidence interval.

**Figure 6:**
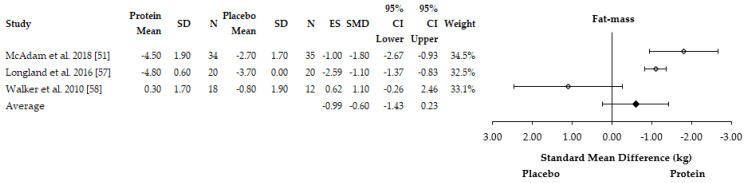
Forest plot of the studies which assessed the effects of protein supplementation on fat-mass changes in response to concurrent training. SD = standard deviation, *N* = sample size, ES = effect size, SMD = standard mean difference, CI = confidence interval.

Fat-free mass (FFM) was shown to increase to a greater extent with protein supplementation over an eight-week training period in military recruits (protein: +0.7 ± 1.2 kg; placebo 0.0 ± 0.9 kg, *p* < 0.05) [58]. Similarly, FFM was also shown to increase to a greater extent in female basketball players over an eight-week period (protein: +1.4 kg; placebo: +0.4 kg, *p* = 0.025) [56]. McAdam et al. [51] also observed greater reductions in fat-mass over an eight-week period in military recruits with protein supplementation compared to a placebo (protein: −4.5 kg; placebo: −2.7 kg, *p* = 0.04) after controlling for initial fat-mass [51]. A trend for greater reductions in fat-mass (protein: −1.0 ± 0.3 kg; placebo: −0.3 ± 0.4 kg, *p* > 0.05) and gains in FFM (protein: +2.4 ± 0.3 kg; placebo: +1.9 ± 0.3 kg, *p* > 0.05) were reported by Ormsbee et al. [55]. However, significant differences were observed only at three months into the six-month concurrent training intervention for gains in FFM (protein:+2.6 ± 0.2 kg; placebo: 1.7 ± 0.3 kg, *p* = 0.02) in sedentary men and women. Longland et al. [57] reported greater reductions in fat-mass (protein: −4.8 ± 1.6 kg; placebo: −3.5 ± 1.4 kg, *p* < 0.05) and gains in FFM (+1.2 ± 1.0 vs. +0.1 ± 1.0 kg, *p* < 0.05) with protein supplementation compared to a placebo over a four-week period. Conversely, no effect of leucine supplementation was reported after six-weeks on fat mass (body fat percentage) changes [53]. In total, five out of six studies reported a beneficial impact of protein supplementation on body composition adaptations. Four studies were included in the meta-analysis for changes in FFM [51,55,57,58,59] whereas three studies were included in the meta-analysis for fat-mass [51,57,58]. One study was excluded from the FFM adaptations analysis due to no SD being reported [56] and one study was excluded from the fat-mass adaptations analysis due to body fat percentage being reported [53]. The meta-analysis found that protein supplementation enhanced gains in FFM (ES = 6.29, SMD = +0.75 kg, 95% CI = 0.44–1.06 kg) (Figure 5). The meta-analysis also found protein supplementation to enhance reductions in fat-mass compared to placebo (ES = −0.99, SMD = 0.60 kg, 95% CI = −1.20–0.45 kg) (Figure 6). The individual study effect sizes for FFM and fat-mass adaptations can be found in Supplementary Figures S4 and S5, respectively. There was considerable heterogeneity between studies for FFM (I^2^ = 98%) and fat-mass adaptations (I^2^ = 91%). 

#### 3.4.3. Muscle Recovery Adaptations

Perceived muscle soreness after a six-mile hike was reduced by 7% with post-exercise protein supplementation compared to increases of 10% and 16% in the placebo and control conditions, respectively (*p* < 0.05) [61]. The remaining studies found no significant difference between protein and placebo conditions for the recovery of muscle function [60], muscle damage [59] or both [52]. Blacker et al. [60] reported no effect of protein compared to CHO on muscle function recovery. At 48 hours post-exercise, knee extensor isometric force was reduced by 10 ± 10% for the low caloric placebo condition (*p* = 0.008) but had returned to baseline in the CHO (*p* = 0.199) and protein condition (*p* = 0.099). At 72 h post-exercise, participants in the placebo condition returned to baseline (*p* = 0.145), whereas both the CHO (*p* = 0.457) and protein conditions (*p* = 0.731) remained at baseline at 48 h post-exercise. Only one study assessed the impact of protein supplementation on markers of exercise induced muscle damage and inflammation [59]. It was found that there were no differences between protein and isocaloric placebo conditions for changes in blood concentrations of cortisol (placebo: −0.79 ± 0.89; protein: 1.39 ± 1.08 µg·dL^−1^, *p* = 0.160), C-reactive protein (placebo: 0.13 ± 0.77; protein: 0.99 ± 0.16 mg·L^−1^, *p* = 0.305), creatine kinase (placebo: 278.65 ± 50.23; protein: 422.18 ± 149.87 U·L^−1^, *p* = 0.722) or aldolase (placebo: 2.06 ± 0.46; protein: 1.98 ± 0.91 U·L^−1^, *p* = 0.704). Based on the limited number of studies and available data, it was not possible to complete a meta-analysis of studies assessing the effect of protein supplementation on muscle recovery adaptations. These limitations include the SD not being reported [61] and different outcome measures, such as muscle damage [59], muscle function [52,60] and muscle soreness [61].

## 4. Discussion

This review identified 11 studies which investigated the effects of protein supplementation on exercise performance, body composition and muscle recovery adaptations to concurrent exercise training compared to a placebo in healthy adults, confirming the need for more work in this area. The key findings from the literature that met our inclusion criteria demonstrated that protein supplementation had a large effect on muscle strength and FFM adaptations to concurrent exercise training. There was limited evidence to suggest that protein supplementation can support aerobic endurance and muscle recovery adaptations. 

### 4.1. Muscle Strength and Body Composition Adaptations

Longland et al. [57] reported no impact of protein supplementation on muscle strength adaptations despite a greater increase in FFM compared to a placebo condition. This was the only study to purposely induce a negative energy balance while participants consumed a total protein intake of 2.4 g·kg^−1^·day^−1^. The study duration (four weeks) may have been too short for differences in strength development to be detected, particularly as protein supplementation is suggested to promote gains in FFM and muscle strength as the duration of training increases [62]. Forbes and Bell [54] also reported no effect of an additional 2.0–2.4 g·kg^−1^·day^−1^ of protein on muscle strength and body composition adaptations over a six-week period (Table 2). It may be that the findings were also confounded by the intervention duration, given that it was shorter than each study reporting a positive effect of protein supplementation. The results were analysed by sex and the small sample size (15 women and 16 men) may have also limited the statistical findings as acknowledged by the study authors. Furthermore, the participants in the control condition consumed 1.2–1.4 g·kg^−1^·day^−1^ of protein, which may have been adequate to meet the demands of training, however, with no measure of nitrogen balance or protein turnover, this cannot be confirmed. The final study which observed no effect of protein supplementation on body composition adaptations may have had low total daily protein intakes (0.85 g·kg^−1^·day^−1^) [53].

The studies that reported a positive effect of protein supplementation on muscle strength and body composition adaptations provided protein to participants immediately post-exercise [51,55,56,57,58]. This likely maximised myofibrillar protein synthesis in response to concurrent training [33,35,38,39] and modulated muscle strength and FFM adaptations [63,64]. Promoting MPS post-exercise is an important factor at enhancing skeletal muscle remodelling and adaptation [39,42,64,65]. Subsequently, this could have attenuated the interference effect of endurance training on strength adaptations [35,66] by promoting the activation of mTOR and inhibiting the activation of the tuberous sclerosis complex [27,28]. Skeletal muscle is sensitive to protein feeding for 24 hours post-exercise and thus, consuming protein in 20–40 g doses evenly throughout the day is recommended [64,67,68]. More recently, it has been shown that consuming protein prior to sleep also augments MPS throughout the night [69]. Consuming protein prior to sleep, and subsequently increasing total daily protein intake may be advantageous at optimising MPS responses and supporting muscle strength and body composition adaptations when undertaking concurrent training [70].Nevertheless, it should be acknowledged that acute changes in MPS does not necessarily predict changes in muscle strength and FFM [71]. Instead it is likely the chronic and repetitive changes in MPS and muscle protein breakdown (MPB) which contribute to these [65]. Two studies that reported a greater increase in muscle strength reported a larger reduction in fat-mass with protein intakes ≥2.2 g·kg^−1^·day^−1^ compared to a placebo [51,55]. This suggests that individuals undergoing arduous concurrent training may benefit from protein intakes higher than the current recommendation of 1.8–2.2 g·kg^−1^·day^−1^ [72]. Mechanistically, it is speculative as to how an elevated protein intake promoted a greater loss in fat-mass, but previous work suggests that the greater thermic effect of protein may play a key role [73]. However, despite similar daily energy intakes between groups in both studies, neither included a measure of energy expenditure, and therefore, it is unclear if participants were in energy balance. Consuming a protein intake >2.2 g·kg^−1^·day^−1^ and possibly higher than 3.0 g·kg^−1^·day^−1^ while restricting energy intake has been suggested to maximise the loss of fat-mass and promote the maintenance of FFM [72]. It is unclear if the greater reduction of fat-mass promoted greater improvements in muscle strength performance in studies included in this review [51,55]. More work is needed to better determine the impact of protein intakes higher than the current recommendations (1.7–2.2 g·kg^−1^·day^−1^) on body composition adaptations, and how this may influence exercise performance in individuals undergoing arduous concurrent training. The studies identified in this review suggest that protein supplementation may be an effective strategy at augmenting muscle strength and body composition adaptations in healthy adults undertaking concurrent training. It is likely that this effect is facilitated by maximising the MPS post-exercise and attenuating the potential interference effect of endurance training on muscle strength and FFM adaptations. However, more work which includes measures of nitrogen balance or protein turnover are needed to confirm this. Furthermore, future work should also consider factors such as the timing of protein intake around exercise, energy intake/expenditure, and the duration of the training intervention. 

### 4.2. Aerobic Adaptations

Protein requirements are elevated in endurance athletes to 1.6–1.8 g·kg^−1^·day^−1^ [74] but may be higher (1.7–2.2 g·kg^−1^·day^−1^) during periods of intense and/or high volume training [72]. Protein feeding has been shown to facilitate recovery and performance adaptations to endurance training [17,64]. Nonetheless, the effects of protein supplementation on aerobic performance adaptations during an arduous concurrent training programme are unknown. Similar improvements in run time performance was observed in military recruits with total daily protein intake of 2.8 ± 0.5 and 1.6 ± 0.4 g·kg^−1^·day^−1^ in the protein and placebo conditions, respectively [51]. As such, the dietary protein requirements to facilitate endurance-based adaptations were likely met in both groups, therefore, between groups differences were not observed. This suggests that to promote endurance-based performance adaptations, additional protein intake is not warranted when total habitual intake is ≥1.6 g·kg^−1^·day^−1^. Walker et al. [58] also observed no between group differences in run time performance in military recruits supplemented with whey protein or CHO for eight-weeks. However, the total daily protein intakes were not reported, and it is unknown if protein requirements were met. In contrast, one study found leucine supplementation (45 mg·kg^−1^·day^−1^) for six-weeks improved exercise time to exhaustion in canoeists [53]. The reported total daily protein intake was 0.85 ± 0.06 g·kg^−1^·day^−1^ and 0.85 ± 0.05 g·kg^−1^·day^−1^ in the protein and placebo groups, therefore, participants likely benefited from the elevated leucine intake given that this amount of protein per day is much lower than the recommended amount [72,74]. Leucine is the key amino acid which stimulates MPS through the mTOR pathway [39,75], but given that the other essential amino acids are required to support this process [36,39], it is unclear how leucine supplementation improved exercise time to exhaustion. The results of the meta-analysis suggest that there is limited evidence to support the use of protein supplementation for aerobic endurance adaptations in response to concurrent training compared to placebo. However, based on the limited number of studies identified, more work is needed to confirm this. 

### 4.3. Muscle Recovery Adaptations

Protein supplementation has been shown to improve muscle function recovery following resistance training [19] and other modes of exercise including cycling, running, eccentric exercise and resistance training [62], yet no review has evaluated the effects following arduous concurrent exercise training specifically. Protein consumption in close proximity to exercise prevents a decrease in myogenin messenger RNA expression, which can accelerate the remodelling and recovery of skeletal muscle [76]. Specifically, leucine may be a key component at initiating this process post-exercise through the activation of mTOR and MPS [39,75]. One study found post-exercise protein ingestion reduced muscle soreness in U.S. Marines following a loaded march [61]. However, Flakoll et al. [61] failed to provide the total daily protein intake, which therefore, limits our understanding of the impact of protein supplementation specifically on recovery adaptations [77]. The remaining studies all failed to find an effect of protein supplementation on muscle recovery in the days following arduous concurrent exercise [52,59,60]. Specifically, no impact was observed on markers of muscle damage [59], soreness [52] or function [52,60]. Jimenez-Flores et al. [59] observed no differences in markers of muscle inflammation or damage between protein and placebo conditions. However, some of the markers which were chosen may be questionable. For example, cortisol is a stress hormone [78,79] which can indicate changes in whole-body catabolism [79]. C-reactive protein is a marker of whole-body inflammation and is not necessarily specific to skeletal muscle [78]. The data also suggest a large inter-participant variability, which is a known limitation of such markers, particularly creatine kinase [79].

Eccentric exercise initiates a chain of events which leads to myofibrillar damage, degradation of structural proteins and membrane damage, thus inhibiting muscle function especially if individuals are unaccustomed to the exercise bout [80]. The participants in the study by Eddens et al. [52] completed a bout of concurrent endurance and eccentric exercise. The participants consumed a similar total daily protein intake, which corresponds to current recommendations [64]. As such, it is possible that the additional protein consumed post-exercise in the experimental group did not accelerate muscle recovery, due to protein requirements already being met by the participants. It was acknowledged by Eddens et al. [52] that the decrement in muscle function over the 24 h post-exercise was ~15%, which is lower than that observed with other eccentric exercise protocols, with decrements of between 10–65% reported elsewhere [81]. Therefore, it cannot be excluded that the muscle damaging protocol may not have been arduous enough, which might explain the lack of statistical difference between conditions [52]. Blacker et al. [60] found no statistically significant difference between protein and placebo conditions on acute muscle function recovery following arduous concurrent exercise. However, both supplement conditions accelerated recovery of muscle function compared to the control condition. Similarly, participants consumed a standardised total daily amount of protein (0.9 ± 0.3 g·kg^−1^·day^−1^) across conditions. Although the amount of protein is lower than the current general recommendations (1.2–2.0 g·kg^−1^·day^−1^) [64], no effect of protein supplementation post-exercise was observed by Blacker et al. [60] when compared to an isocaloric placebo [60]. Jimenez-Flores et al. [59] found no impact of protein supplementation compared to an isocaloric placebo on markers of muscle damage following arduous concurrent exercise, however, high-variability between study participants was observed and likely influenced the ability to detect statistical differences [59]. However, unlike Blacker et al. [60], there was no control group, therefore, it is unknown if the additional energy intake accelerated muscle recovery [59]. The limited number of studies and the differences between methodologies and outcome measures make it difficult to determine whether protein supplementation does improve muscle recovery, thus warranting further research. Additionally, due to the lack of women included in studies to date, future research is needed in women to examine the impact of protein on muscle recovery, particularly given the known difference in the rate of muscle function recovery post-exercise between men and women [82]. 

### 4.4. Limitations

Only one study identified in this review included a measure of protein balance or turnover [57]. Therefore, it is unknown if participants were in a positive protein balance before, during or after the interventions in the remaining studies. Including a measure of protein requirements in future studies can allow for a better understanding of how much additional protein is potentially needed during arduous concurrent training by estimating changes in MPS and whole-body protein balance. The majority of studies failed to include markers of skeletal muscle damage or inflammation when focusing on muscle recovery and therefore, the mechanisms of the effects observed are speculative. The control of dietary intake is critical for comparison between studies involving nutritional interventions. Four studies failed to report the total daily protein intake during the intervention [56,58,59,61], making the comparisons between studies even more challenging, given that this is considered more important than the timing of protein intake [71,83]. It is recommended that these methodological considerations be factored into future studies aimed at investigating the influence of protein supplementation on arduous concurrent training adaptations. The heterogeneity of the meta-analysis results should be acknowledged as this may make it difficult to apply these findings to a specific population. Nonetheless, the findings of this review infer that protein supplementation can support muscle strength, aerobic endurance, and body composition adaptations during concurrent training. However, more population specific randomised controlled trials (RCTs) are needed to build upon these findings. 

### 4.5. Military Research Applications

Five of the eleven studies included in this review quantified the effects of protein supplementation in those during military training, or in response to a military training-based activity [51,58,59,60,61]. Given the potential for concurrent endurance training to inhibit muscle strength adaptations [27,28], military recruits may be one population who can benefit from strategies which aim to promote gains in FFM and muscle strength during arduous concurrent military training. The findings of this systematic review suggest that protein supplementation may be an effective strategy to support body composition and muscle strength development. However, to better understand the effects protein supplementation has on adaptation and performance outcomes of military recruits, additional population specific RCTs are needed. Future RCTs should consider investigating the effects of elevated protein intakes on training adaptations during arduous military training, including muscle strength, body composition and muscle recovery. Additionally, future work may want to consider adaptations not included in this review, such as bone adaptations, given that bone health and stress fracture incidence are important areas of military research [84,85,86] and which protein supplementation may be able to support [70,87]. The lack of women studied to date in this area also highlights a gap in the current literature. Therefore, future work should aim to include data in women since they now take-up more arduous (ground close combat) roles in the military [88]. Furthermore, including measures of protein metabolism, such as nitrogen balance and protein turnover, in future work should be considered as a means of better understanding the effects of protein supplementation during military recruit training. 

## 5. Conclusions

This is the first systematic review and meta-analysis to investigate the effects of protein supplementation on arduous concurrent training adaptations. Based on the limited number of studies identified, more work in this area is clearly warranted, particularly given the importance of developing aerobic fitness and muscle strength concurrently in many exercising populations. The findings of this review suggest that protein supplementation may be an effective strategy at supporting lean-mass accretion and muscle strength adaptations in healthy adults, whilst considering the impact that training programme duration, total energy and protein intake of participants has on outcome measures. From the existing literature, it is reasonable to recommend that individuals aim for total daily protein intakes between 1.7 and 2.2 g·kg^−1^·day^−1^ whilst ingesting 20–40 g of protein immediately post-exercise to maximise MPS and support muscle strength and FFM adaptations. However, the disassociation between MPS and chronic physiological adaptations should be acknowledged. Based on the novel data included in this review, subsequent research may consider investigating the potential benefits of higher total daily protein intakes during arduous concurrent training on adaptation and performance outcomes. 

## Figures and Tables

**Table 1:** The individual and mean reviewer quality assessment scores for each study.

Study	Reviewer 1	Reviewer 2	Mean	Included
McAdam et al. [51]	4.00	3.70	3.85	Y
Eddens et al. [52]	3.60	3.30	3.45	Y
Crowe, Weatherson and Bowden [53]	4.00	3.40	3.70	Y
Forbes and Bell [54]	3.80	3.20	3.50	Y
Ormsbee et al. [55]	3.80	3.40	3.60	Y
Taylor et al. [56]	4.00	4.00	4.00	Y
Longland et al. [57]	4.00	3.50	3.75	Y
Walker et al. [58]	4.00	3.90	3.95	Y
Jimenez-Flores et al. [59]	3.30	2.90	3.10	Y
Blacker et al. [60]	3.40	3.00	3.20	Y
Flakoll et al. [61]	4.00	3.80	3.90	Y

Yes = Y; 1 = poor, 2 = fair,3 = good,4 = excellent.

**Table 2:** The impact of protein on performance and body composition during concurrent exercise training.

Study	Sample	Age	Total Protein Intake	Intervention	Supplement Type & Dose
McAdam et al. [51]	69 male U.S. Army recruits.	19 ± 1 years	2.8 ± 0.5 & 1.6 ± 0.4 g·kg^−1^·d^−1^ in PRO and PLA.	8-week U.S. Army Initial Entry Training.	38.6 g WP or isocaloric PLA post-exercise in AM & prior to sleep.
Crowe, Weatherson and Bowden [53]	10 male, 3 female trained canoeists.	32 ± 2 years	0.85 ± 0.06 & 0.85 ± 0.05 g·kg^−1^·day^−1^ in PRO & PLA.	6-weeks endurance & resistance training.	45 mg·kg^−1^·day^−1^ leucine or PLA.
Ormsbee et al. [55]	26 sedentary men and 25 sedentary women.	21± 1 years & 20 ± 1 years in PRO & PLA.	2.2 ± 0.1 & 1.1 ± 0.1 g·kg^−1^·day^−1^ for the PRO & PLA groups.	6-month endurance & resistance training.	42 g PRO or isocaloric PLA consumed immediately post-exercise & 8–12 h later.
Taylor et al. [56]	16 female intermittent sport athletes.	20 ± 2 years	Not measured.	8-week endurance & resistance training.	24 g pre-and-post-exercise.
Longland et al. [57]	40 recreationally active men.	23 ± 2 years	2.4 & 1.2 g·kg^−1^·day^−1^ for the PRO & PLA groups.	4-weeks endurance & resistance training with an energy deficit	50 g WP or CHO drink given post-exercise to PRO & PLA groups.
Walker et al. [56]	30 U.S. Air force men.	26 ± 9 years	Not measured.	8-week U.S. Air force training.	20 g WP or isocaloric PLA post-exercise.
Forbes and Bell [54]	15 healthy women & 16 men.	Women: 27 ± 4 years, men: 26 ± 3 years	PLA (men = 1.4 ± 0.4 g·kg^−1^·day^−1^, women= 1.2 ± 0.2 g·kg^−1^·day^−1^), PRO (men = 3.8 ± 0.4 g·kg^−1^·day^−1^, women= 3.2 ± 0.3 g·kg^−1^·day^−1^).	6-weeks endurance & resistance training.	2.0 and 2.4 g·kg^−1^·day^−1^ WP for women & men.

Data reported as mean ± standard deviation where possible. U.S. = United states, g·kg^−1^·day^−1^ = grams per kilogram of body mass per day, PLA = placebo, PRO = protein, WP = whey protein, CHO = carbohydrate, CON = control, AM = morning.

**Table 3:** Concurrent exercise training and the impact of protein on muscle recovery.

Study	Sample	Age	Total Protein Intake	Intervention	Supplement Type & Dose
Eddens et al. [52]	24 male cyclists.	PRO = 27 ± 3 years; PLA = 28 ± 5 years; CHO = 26 ± 5 years	PRO = 1.2 ± 0.6 g·kg^−1^·day^−1^; PLA = 1.2 ± 0.6 g·kg^−1^·day^−1^; CHO = 1.2 ± 0.7 g·kg^−1^·day^−1^	Single concurrent exercise event (high-intensity cycling followed by 100 box jumps).	20 g WP, isocaloric CHO or low-calorific PLA post-exercise.
Jimenez-Flores et al. [59]	33 healthy men and 2 healthy women.	21 ± 1 years & 21 ± 1 years in PLA & PRO groups	Not measured.	4-day loaded (13.2–26.4 kg) mountain skirmish.	25 g protein bar or isocaloric CHO bar post-exercise.
Blacker et al. [60]	10 healthy men.	28 ± 9 years	* 0.9 ± 0.3 g·kg^−1^·day^−1^, in the PLA, CHO & PRO.	3 days post-load (25 kg) carriage exercise.	36 g PRO, 32 g CHO or low-calorie PLA post-exercise.
Flakoll et al. [61]	387 male U.S. Marine recruits.	19 ± 1 years	Not measured.	Single day loaded march hike.	PLA = 0 g CHO, 0 g PRO, 0 g fat; CON = 0 g PRO, 8 g CHO and 3 g fat; PRO = 10 g PRO, 8 g CHO and 3 g fat. Participants who weighed <81.8 kg received one portion and those weighing >81.8 kg received two portions post-exercise.

Data reported as mean ± standard deviation. U.S. = United States, g·kg^−1^·day^−1^ = grams per kilogram of body mass per day, PLA = placebo, PRO = protein, WP = whey protein, CHO = carbohydrate, CON = control. * maximum across three timepoints.

## Data Availability

See Supplementary Materials. Raw data available on request.

## Supplementary Materials

The following are available online at https://www.mdpi.com/article/10.3390/nu13051416/s1, Figure S1: Funnel plot of the comparison of the effect of protein supplementation vs. placebo on muscle strength, aerobic endurance, FFM and fat-mass adaptations. All studies are included. Figure S2: Forest plot showing the effect sizes of the studies which assessed the effects of protein supplementation on muscle strength adaptations. Figure S3: Forest plot showing the effect sizes of the studies which assessed the effects of protein supplementation on aerobic endurance adaptations. Figure S4: Forest plot showing the effect sizes of the studies which assessed the effects of protein supplementation on fat-free mass (FFM) changes in response to concurrent training. Figure S5: Forest plot showing the effect sizes of the studies which assessed the effects of protein supplementation on fat-mass changes in response to concurrent training.

## References

[1] Wilson J.M., Marin P.J., Rhea M.R., Wilson S.M., Loenneke J.P., Anderson J.C. (2012). Concurrent Training. J. Strength Cond. Res..

[2] Nader G.A. (2006). Concurrent Strength and Endurance Training. Med. Sci. Sports Exerc..

[3] Claudino J.G., Gabbett T.J., Bourgeois F., Souza H.D.S., Miranda R.C., Mezêncio B., Soncin R., Filho C.A.C., Bottaro M., Hernandez A.J. (2018). CrossFit Overview: Systematic Review and Meta-analysis. Sports Med.-Open.

[4] Bonnici D.C., Greig M., Akubat I., Sparks S.A., Bentley D., Mc Naughton L.R. (2019). Nutrition in Soccer: A Brief Review of the Issues and Solutions. J. Sci. Sport Exerc..

[5] Bradley W., Cavanagh B., Douglas W., Donovan T.F., Morton J.P., Close G.L. (2014). Quantification of Training Load, Energy Intake, and Physiological Adaptations during a Rugby Preseason: A Case Study from an Elite European Rugby Union Squad. J. Strength Cond. Res..

[6] Calleja-González J., Mielgo-Ayuso J., Sampaio J., Delextrat A., Ostojic S.M., Marques-Jiménez D., Arratibel I., Sánchez-Ureña B., Dupont G., Schelling X. (2018). Brief ideas about evidence-based recovery in team sports. J. Exerc. Rehabil..

[7] O’Leary T., Saunders S., McGuire S., Kefyalew S., Venables M., Izard R. (2017). Sex differences in physical performance and body composition adaptations to British Army basic military training. J. Sci. Med. Sport.

[8] Drain J.R., Sampson J.A., Billing D.C., Burley S.D., Linnane D.M., Groeller H. (2015). The Effectiveness of Basic Military Training to Improve Functional Lifting Strength in New Recruits. J. Strength Cond. Res..

[9] Richmond V.L., Carter J.M., Wilkinson D.M., Horner F.E., Rayson M.P., Wright A., Bilzon J.L. (2012). Comparison of the Physical Demands of Single-Sex Training for Male and Female Recruits in the British Army. Mil. Med..

[10] Richmond V.L., Horner F.E., Wilkinson D.M., Rayson M.P., Wright A., Izard R. (2014). Energy Balance and Physical Demands During an 8-Week Arduous Military Training Course. Mil. Med..

[11] Blacker S.D., Wilkinson D.M., Rayson M.P. (2009). Gender Differences in the Physical Demands of British Army Recruit Training. Mil. Med..

[12] Sharma J., Heagerty R., Dalal S., Banerjee B., Booker T. (2018). Risk Factors Associated With Musculoskeletal Injury: A Prospective Study of British Infantry Recruits. Curr. Rheumatol. Rev..

[13] Wardle S.L., Greeves J.P. (2017). Mitigating the risk of musculoskeletal injury: A systematic review of the most effective injury prevention strategies for military personnel. J. Sci. Med. Sport.

[14] O’Leary T.J., Wardle S.L., Greeves J.P. (2020). Energy Deficiency in Soldiers: The Risk of the Athlete Triad and Relative Energy Deficiency in Sport Syndromes in the Military. Front. Nutr..

[15] Chapman S., Rawcliffe A.J., Izard R., Jacka K., Tyson H., Smith L., Roberts J. (2020). Dietary Intake and Nitrogen Balance in British Army Infantry Recruits Undergoing Basic Training. Nutrients.

[16] Cermak N.M., Res P.T., De Groot L.C.P.G.M., Saris W.H.M., Van Loon L.J.C. (2012). Protein supplementation augments the adaptive response of skeletal muscle to resistance-type exercise training: A meta-analysis. Am. J. Clin. Nutr..

[17] Lin Y.-N., Tseng T.-T., Knuiman P., Chan W.P., Wu S.-H., Tsai C.-L., Hsu C.-Y. (2020). Protein supplementation increases adaptations to endurance training: A systematic review and meta-analysis. Clin. Nutr..

[18] Morton R.W., Murphy K.T., McKellar S.R., Schoenfeld B.J., Henselmans M., Helms E., Aragon A., Devries M.C., Banfield L., Krieger J.W. (2018). A systematic review, meta-analysis and meta-regression of the effect of protein supplementation on resistance training-induced gains in muscle mass and strength in healthy adults. Br. J. Sports Med..

[19] Davies R.W., Carson B.P., Jakeman P.M. (2018). The Effect of Whey Protein Supplementation on the Temporal Recovery of Muscle Function Following Resistance Training: A Systematic Review and Meta-Analysis. Nutrients.

[20] Wilkinson S.B., Phillips S.M., Atherton P.J., Patel R., Yarasheski K.E., Tarnopolsky M.A., Rennie M.J. (2008). Differential effects of resistance and endurance exercise in the fed state on signalling molecule phosphorylation and protein synthesis in human muscle. J. Physiol..

[21] Egan B., Zierath J.R. (2013). Exercise Metabolism and the Molecular Regulation of Skeletal Muscle Adaptation. Cell Metab..

[22] Craig B.W., Lucas J., Pohlman R., Stelling H. (1991). The Effects of Running, Weightlifting and a Combination of Both on Growth Hormone Release. J. Strength Cond. Res..

[23] Hennessy L.C., Watson A.W.S. (1994). The Interference Effects of Training for Strength and Endurance Simultaneously. J. Strength Cond. Res..

[24] Kraemer W.J., Patton J.F., Gordon S.E., Harman E.A., Deschenes M.R., Reynolds K., Newton R.U., Triplett N.T., Dziados J.E. (1995). Compatibility of high-intensity strength and endurance training on hormonal and skeletal muscle adaptations. J. Appl. Physiol..

[25] Fyfe J.J., Bishop D.J., Bartlett J.D., Hanson E.D., Anderson M.J., Garnham A.P., Stepto N.K. (2018). Enhanced skeletal muscle ribosome biogenesis, yet attenuated mTORC1 and ribosome biogenesis-related signalling, following short-term concurrent versus single-mode resistance training. Sci. Rep..

[26] Fyfe J.J., Bartlett J.D., Hanson E.D., Stepto N.K., Bishop D.J. (2016). Endurance Training Intensity Does Not Mediate Interference to Maximal Lower-Body Strength Gain during Short-Term Concurrent Training. Front. Physiol..

[27] Baar K. (2006). Training for Endurance and Strength: Lessons from Cell Signaling. Med. Sci. Sport Exerc..

[28] Baar K. (2014). Using molecular biology to maximize concurrent training. Sports Med..

[29] Lundberg T.R., Fernandez-Gonzalo R., Gustafsson T., Tesch P.A. (2013). Aerobic exercise does not compromise muscle hypertrophy response to short-term resistance training. J. Appl. Physiol..

[30] De Souza E.O., Roschel H., Brum P.C., Bacurau A.N., Ferreira J.B., Aoki M.S., Neves M., Aihara A.Y., Ugrinowitsch C., Tricoli V. (2012). Molecular Adaptations to Concurrent Training. Int. J. Sports Med..

[31] Lundberg T.R., Fernandez-Gonzalo R., Tesch P.A. (2014). Exercise-induced AMPK activation does not interfere with muscle hypertrophy in response to resistance training in men. J. Appl. Physiol..

[32] Lantier L., Fentz J., Mounier R., Leclerc J., Treebak J.T., Pehmøller C., Sanz N., Sakakibara I., Saint-Amand E., Rimbaud S. (2014). AMPK controls exercise endurance, mitochondrial oxidative capacity, and skeletal muscle integrity. FASEB J..

[33] Macnaughton L.S., Wardle S.L., Witard O.C., McGlory C., Hamilton D.L., Jeromson S., Lawrence C.E., Wallis G.A., Tipton K.D. (2016). The response of muscle protein synthesis following whole-body resistance exercise is greater following 40 g than 20 g of ingested whey protein. Physiol. Rep..

[34] Donges C.E., Burd N.A., Duffield R., Smith G.C., West D.W.D., Short M.J., MacKenzie R., Plank L.D., Shepherd P.R., Phillips S.M. (2012). Concurrent resistance and aerobic exercise stimulates both myofibrillar and mitochondrial protein synthesis in sedentary middle-aged men. J. Appl. Physiol..

[35] Camera D.M., West D.W.D., Phillips S.M., Rerecich T., Stellingwerff T., Hawley J.A., Coffey V.G. (2015). Protein Ingestion Increases Myofibrillar Protein Synthesis after Concurrent Exercise. Med. Sci. Sports Exerc..

[36] Pasiakos S.M. (2012). Exercise and Amino Acid Anabolic Cell Signaling and the Regulation of Skeletal Muscle Mass. Nutrients.

[37] Roberts J., Zinchenko A., Suckling C., Smith L., Johnstone J., Henselmans M. (2017). The short-term effect of high versus moderate protein intake on recovery after strength training in resistance-trained individuals. J. Int. Soc. Sports Nutr..

[38] Tipton K.D. (2008). Protein for adaptations to exercise training. Eur. J. Sport Sci..

[39] Witard O.C., Wardle S.L., Macnaughton L.S., Hodgson A.B., Tipton K.D. (2016). Protein Considerations for Optimising Skeletal Muscle Mass in Healthy Young and Older Adults. Nutrients.

[40] Tipton K.D., Sharp C.P. (2005). The response of intracellular signaling and muscle-protein metabolism to nutrition and exercise. Eur. J. Sport Sci..

[41] Tipton K.D., Wolfe R.R. (2004). Protein and amino acids for athletes. J. Sports Sci..

[42] Phillips S.M., Hartman J.W., Wilkinson S.B. (2005). Dietary Protein to Support Anabolism with Resistance Exercise in Young Men. J. Am. Coll. Nutr..

[43] Phillips S.M., Van Loon L.J. (2011). Dietary protein for athletes: From requirements to optimum adaptation. J. Sports Sci..

[44] McLellan T.M. (2013). Protein Supplementation for Military Personnel: A Review of the Mechanisms and Performance Outcomes. J. Nutr..

[45] Moher D., Liberati A., Tetzlaff J., Altman D.G., The PRISMA Group (2009). Preferred reporting items for systematic reviews and meta-analyses: The PRISMA statement. PLoS Med..

[46] Methley A.M., Campbell S., Chew-Graham C., McNally R., Cheraghi-Sohi S. (2014). PICO, PICOS and SPIDER: A comparison study of specificity and sensitivity in three search tools for qualitative systematic reviews. BMC Health Serv. Res..

[47] Blacker S.D., Wilkinson D.M., Bilzon J.L., Rayson M.P. (2008). Risk Factors for Training Injuries among British Army Recruits. Mil. Med..

[48] Needleman I.G. (2002). A guide to systematic reviews. J. Clin. Periodontol..

[49] Terwee C.B., Mokkink L.B., Knol D.L., Ostelo R.W.J.G., Bouter L.M., De Vet H.C.W. (2011). Rating the methodological quality in systematic reviews of studies on measurement properties: A scoring system for the COSMIN checklist. Qual. Life Res..

[50] Cohen J. (2007). A power primer. Tutor. Quant. Methods Psychol..

[51] McAdam J.S., McGinnis K.D., Beck D.T., Haun C.T., Romero M.A., Mumford P.W., Roberson P.A., Young K.C., Lohse K.R., Lockwood C.M. (2018). Effect of Whey Protein Supplementation on Physical Performance and Body Composition in Army Initial Entry Training Soldiers. Nutrients.

[52] Eddens L., Browne S., Stevenson E.J., Sanderson B., Van Someren K., Howatson G. (2017). The efficacy of protein supplementation during recovery from muscle-damaging concurrent exercise. Appl. Physiol. Nutr. Metab..

[53] Crowe M.J., Weatherson J.N., Bowden B.F. (2005). Effects of dietary leucine supplementation on exercise performance. Graefe’s Arch. Clin. Exp. Ophthalmol..

[54] Forbes S.C., Bell G.J. (2020). Whey protein isolate or concentrate combined with concurrent training does not augment performance, cardiorespiratory fitness, or strength adaptations. J. Sports Med. Phys. Fit..

[55] Ormsbee M.J., Willingham B.D., Marchant T., Binkley T.L., Specker B.L., Vukovich M.D. (2018). Protein Supplementation During a 6-Month Concurrent Training Program: Effect on Body Composition and Muscular Strength in Sedentary Individuals. Int. J. Sport Nutr. Exerc. Metab..

[56] Taylor L.W., Wilborn C., Roberts M.D., White A.J.P., Dugan M.K. (2016). Eight weeks of pre- and postexercise whey protein supplementation increases lean body mass and improves performance in Division III collegiate female basketball players. Appl. Physiol. Nutr. Metab..

[57] Longland T.M., Oikawa S.Y., Mitchell C.J., Devries M.C., Phillips S.M. (2016). Higher compared with lower dietary protein during an energy deficit combined with intense exercise promotes greater lean mass gain and fat mass loss: A randomized trial. Am. J. Clin. Nutr..

[58] Walker T.B., Smith J., Herrera M., Lebegue B., Pinchak A., Fischer J. (2010). The Influence of 8 Weeks of Whey-Protein and Leucine Supplementation on Physical and Cognitive Performance. Int. J. Sport Nutr. Exerc. Metab..

[59] Jimenez-Flores R., Heick J., Davis S.C., Hall K.G., Schaffner A. (2012). A Comparison of the Effects of a High Carbohydrate vs. a Higher Protein Milk Supplement Following Simulated Mountain Skirmishes. Mil. Med..

[60] Blacker S.D., Williams N.C., Fallowfield J.L., Bilzon J.L., Willems M.E. (2010). Carbohydrate vs protein supplementation for recovery of neuromuscular function following prolonged load carriage. J. Int. Soc. Sports Nutr..

[61] Flakoll P.J., Judy T., Flinn K., Carr C., Flinn S. (2004). Postexercise protein supplementation improves health and muscle soreness during basic military training in marine recruits. J. Appl. Physiol..

[62] Pasiakos S.M., McLellan T.M., Lieberman H.R. (2015). The Effects of Protein Supplements on Muscle Mass, Strength, and Aerobic and Anaerobic Power in Healthy Adults: A Systematic Review. Sports Med..

[63] Witard O.C., Garthe I., Phillips S.M., Philips S.M. (2019). Dietary Protein for Training Adaptation and Body Composition Manipulation in Track and Field Athletes. Int. J. Sport Nutr. Exerc. Metab..

[64] Jäger R., Kerksick C.M., Campbell B.I., Cribb P.J., Wells S.D., Skwiat T.M., Purpura M., Ziegenfuss T.N., Ferrando A.A., Arent S.M. (2017). International Society of Sports Nutrition Position Stand: Protein and exercise. J. Int. Soc. Sports Nutr..

[65] Damas F., Phillips S.M., Libardi C.A., Vechin F.C., Lixandrão M.E., Jannig P.R., Costa L.A.R., Bacurau A.V., Snijders T., Parise G. (2016). Resistance training-induced changes in integrated myofibrillar protein synthesis are related to hypertrophy only after attenuation of muscle damage. J. Physiol..

[66] Pérez-Schindler J., Hamilton D.L., Moore D.R., Baar K., Philp A. (2015). Nutritional strategies to support concurrent training. Eur. J. Sport Sci..

[67] Areta J.L., Burke L.M., Ross M.L., Camera D.M., West D.W.D., Broad E.M., Jeacocke N.A., Moore D.R., Stellingwerff T., Phillips S.M. (2013). Timing and distribution of protein ingestion during prolonged recovery from resistance exercise alters myofibrillar protein synthesis. J. Physiol..

[68] Mamerow M.M., Mettler J.A., English K.L., Casperson S.L., Arentson-Lantz E., Sheffield-Moore M., Layman D.K., Paddon-Jones D. (2014). Dietary Protein Distribution Positively Influences 24-h Muscle Protein Synthesis in Healthy Adults. J. Nutr..

[69] Trommelen J., Van Loon L.J.C. (2016). Pre-Sleep Protein Ingestion to Improve the Skeletal Muscle Adaptive Response to Exercise Training. Nutrients.

[70] Antonio J., Candow D.G., Forbes S.C., Ormsbee M.J., Saracino P.G., Roberts J. (2020). Effects of Dietary Protein on Body Composition in Exercising Individuals. Nutrients.

[71] Reidy P.T., Rasmussen B.B. (2016). Role of Ingested Amino Acids and Protein in the Promotion of Resistance Exercise–Induced Muscle Protein Anabolism. J. Nutr..

[72] Kerksick C.M., Wilborn C.D., Roberts M.D., Smith-Ryan A.E., Kleiner S.M., Jäger R., Collins R., Cooke M., Davis J.N., Galvan E. (2018). ISSN exercise & sports nutrition review update: Research & recommendations. J. Int. Soc. Sports Nutr..

[73] Aragon A.A., Schoenfeld B.J., Wildman R., Kleiner S., VanDusseldorp T., Taylor L., Earnest C.P., Arciero P.J., Wilborn C., Kalman D.S. (2017). International society of sports nutrition position stand: Diets and body composition. J. Int. Soc. Sports Nutr..

[74] Kato H., Suzuki K., Bannai M., Moore D.R. (2016). Protein Requirements Are Elevated in Endurance Athletes after Exercise as Determined by the Indicator Amino Acid Oxidation Method. PLoS ONE.

[75] Aguirre N., Baar K. (2013). The Role of Amino Acids in Skeletal Muscle Adaptation to Exercise. Issues Complementary Feed..

[76] Hulmi J.J., Kovanen V., Selänne H., Kraemer W.J., Häkkinen K., Mero A.A. (2008). Acute and long-term effects of resistance exercise with or without protein ingestion on muscle hypertrophy and gene expression. Amino Acids.

[77] Huecker M., Sarav M., Pearlman M., Laster J. (2019). Protein Supplementation in Sport: Source, Timing, and Intended Benefits. Curr. Nutr. Rep..

[78] Lindsay A., Costello J.T. (2017). Realising the Potential of Urine and Saliva as Diagnostic Tools in Sport and Exercise Medicine. Sports Med..

[79] Lee E.C., Fragala M.S., Kavouras S.A., Queen R.M., Pryor J.L., Casa D.J. (2017). Biomarkers in Sports and Exercise: Tracking Health, Performance, and Recovery in Athletes. J. Strength Cond. Res..

[80] Markus I., Constantini K., Hoffman J.R., Bartolomei S., Gepner Y. (2021). Exercise-induced muscle damage: Mechanism, assessment and nutritional factors to accelerate recovery. Graefe’s Arch. Clin. Exp. Ophthalmol..

[81] Clarkson P.M., Hubal M.J. (2002). Exercise-Induced Muscle Damage in Humans. Am. J. Phys. Med. Rehabil..

[82] O’Leary T.J., Saunders S.C., McGuire S.J., Izard R.M. (2018). Sex differences in neuromuscular fatigability in response to load carriage in the field in British Army recruits. J. Sci. Med. Sport.

[83] Schoenfeld B.B., Aragon A.A., Krieger J.W. (2013). The effect of protein timing on muscle strength and hypertrophy: A meta-analysis. J. Int. Soc. Sports Nutr..

[84] Moran D.S., Heled Y., Arbel Y., Israeli E., Finestone A.S., Evans R.K., Yanovich R. (2012). Dietary intake and stress fractures among elite male combat recruits. J. Int. Soc. Sports Nutr..

[85] Wentz L., Liu P.-Y., Haymes E., Ilich J.Z. (2011). Females Have a Greater Incidence of Stress Fractures Than Males in Both Military and Athletic Populations: A Systemic Review. Mil. Med..

[86] O’Leary T.J., Walsh N.P., Casey A., Izard R.M., Tang J.C.Y., Fraser W.D., Greeves J.P. (2021). Supplementary Energy Increases Bone Formation during Arduous Military Training. Med. Sci. Sports Exerc..

[87] Sale C., Elliott-Sale K.J. (2019). Nutrition and Athlete Bone Health. Sports Med..

[88] O’Leary T.J., Wardle S.L., Rawcliffe A.J., Chapman S., Mole J., Greeves J.P. (2020). Understanding the musculoskeletal injury risk of women in combat: The effect of infantry training and sex on musculoskeletal injury incidence during British Army basic training. BMJ Mil. Health.

