# Supplementary material for: Does Protein Supplementation Support Adaptations to Arduous Concurrent Exercise Training? A Systematic Review and Meta-Analysis with Military Based Applications
Source: Nutrients. 2021 Apr 23;13(5):1416. doi: 10.3390/nu13051416 (PMC8145048; doi:10.3390/nu13051416)
Supplement: Supplementary file 1 [file nutrients-13-01416-s001.zip › nutrients-1170558-supplementary.pdf]

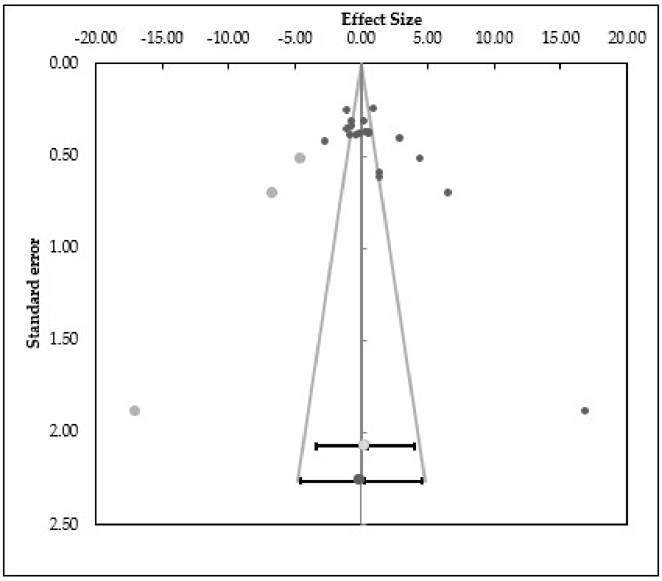

Supplementary Figure S1. Funnel plot of the comparison of the effect of protein supplementation vs. placebo on muscle strength, aerobic endurance, fat free mass (FFM) and fat-mass (FM) adaptations. All studies are included.

Study	Treatment Mean	SD	N	Placebo Mean	SD	N	SMD (ES)	95% CI Lower	95% CI Upper	Weight
Ormsbee et al. 2018 [55]	27.40	2.40	29	15.90	2.80	22	4.46	3.44	5.48	19.5%
Taylor et al. 2016 [56]	4.90	2.10	8	2.30	1.40	6	1.41	0.25	2.58	19.4%
Walker et al. 2010 [58]	3.50	5.20	18	1.30	4.40	12	0.45	-0.29	1.19	20.3%
Longland et al. 2016 [57]	169.00	47.00	20	156.00	32.00	20	0.32	-0.30	0.95	20.6%
Forbes and Bell 2020 [59]	28.70	5.30	21	33.40	8.20	10	-0.74	-1.52	0.03	20.2%
Average							1.18	0.32	2.04	

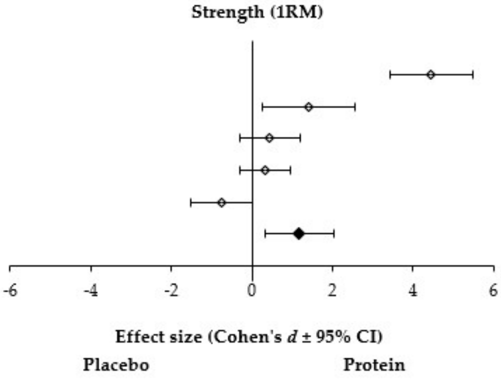

Supplementary Figure S2. Forest plot showing the effect sizes of the studies which assessed the effects of protein supplementation on muscle strength adaptations.

Study	Protein Mean	SD	N	Placebo Mean	SD	N	SMD (ES)	95% CI Lower	95% CI Upper	Weight
Longland et al. 2016 [57]	5.3	2.80	20	6.90	2.00	20	-0.66	-1.29	-0.02	33.7%
Forbes and Bell 2020 [59]	3.40	1.20	21	3.40	3.15	10	0.00	-0.75	0.75	33.3%
Ormsbee et al. 2018 [55]	3.40	0.20	29	2.90	0.10	22	3.03	2.23	3.84	33.1%
Average							0.79	0.06	1.52	

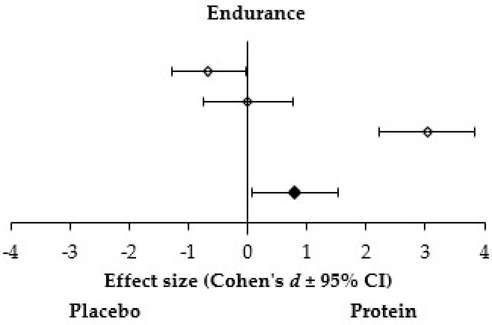

Supplementary Figure S3. Forest plot showing the effect sizes of the studies which assessed the effects of protein supplementation on aerobic endurance adaptations.

Study	Treatment Mean	SD	N	Placebo Mean	SD	N	SMD (ES)	95% CI Lower	95% CI Upper	Weight
McAdam et al. 2018 [51]	4.20	0.40	34	3.60	0.80	35	0.94	0.45	1.44	21.7%
Ormsbee et al. 2018 [55]	2.40	0.10	29	1.90	0.00	22	6.61	5.23	7.99	20.5%
Longland et al. 2016 [57]	1.20	0.10	20	0.00	0.00	20	16.97	13.27	20.67	14.9%
Walker et al. 2010 [58]	0.70	1.20	18	0.00	0.90	12	0.64	-0.11	1.39	21.4%
Average							6.29	4.71	7.87	

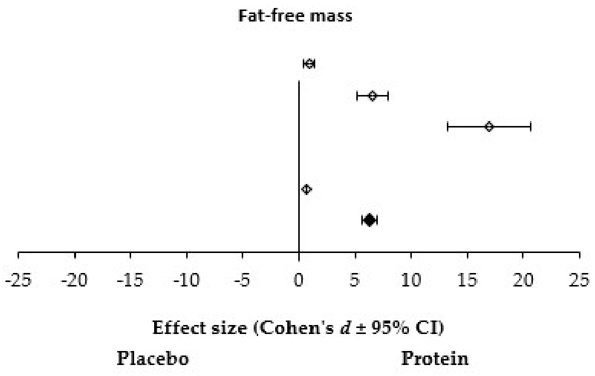

Supplementary Figure S4. Forest plot showing the effect sizes of the studies which assessed the effects of protein supplementation on fat-free mass (FFM) changes in response to concurrent training.

Study	Protein Mean	SD	N	Placebo Mean	SD	N	SMD (ES)	95% CI Lower	95% CI Upper	Weight
McAdam et al. 2018 [51]	-4.50	1.90	34	-2.70	1.70	35	-1.00	-1.50	-0.50	34.5%
Longland et al. 2016 [57]	-4.80	0.60	20	-3.70	0.00	20	-2.59	-3.43	-1.76	32.5%
Walker et al. 2010 [58]	0.30	1.70	18	-0.80	1.90	12	0.62	-0.13	1.36	33.1%
Average							-0.99	-1.68	-0.30	

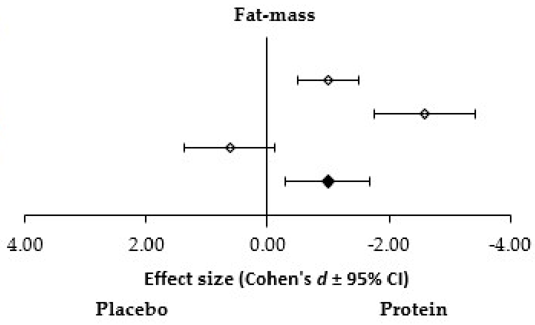

Supplementary Figure S5. Forest plot showing the effect sizes of the studies which assessed the effects of protein supplementation on fat-mass (FM) changes in response to concurrent training.
